# BCI Control of Heuristic Search Algorithms

**DOI:** 10.3389/fninf.2017.00006

**Published:** 2017-01-31

**Authors:** Marc Cavazza, Gabor Aranyi, Fred Charles

**Affiliations:** ^1^School of Engineering and Digital Arts, University of KentCanterbury, UK; ^2^School of Computing, Teesside UniversityMiddlesbrough, UK; ^3^Faculty of Science and Technology, Department of Creative Technology, Bournemouth UniversityPoole, UK

**Keywords:** user interfaces, brain-computer interfaces (BCI), neurofeedback (NF), functional near-infrared spectroscopy (fNIRS), heuristic search

## Abstract

The ability to develop Brain-Computer Interfaces (BCI) to Intelligent Systems would offer new perspectives in terms of human supervision of complex Artificial Intelligence (AI) systems, as well as supporting new types of applications. In this article, we introduce a basic mechanism for the control of heuristic search through fNIRS-based BCI. The rationale is that heuristic search is not only a basic AI mechanism but also one still at the heart of many different AI systems. We investigate how users’ mental disposition can be harnessed to influence the performance of heuristic search algorithm through a mechanism of precision-complexity exchange. From a system perspective, we use weighted variants of the A* algorithm which have an ability to provide faster, albeit suboptimal solutions. We use recent results in affective BCI to capture a BCI signal, which is indicative of a compatible mental disposition in the user. It has been established that Prefrontal Cortex (PFC) asymmetry is strongly correlated to motivational dispositions and results anticipation, such as approach or even risk-taking, and that this asymmetry is amenable to Neurofeedback (NF) control. Since PFC asymmetry is accessible through fNIRS, we designed a BCI paradigm in which users vary their PFC asymmetry through NF during heuristic search tasks, resulting in faster solutions. This is achieved through mapping the PFC asymmetry value onto the dynamic weighting parameter of the weighted A* (WA*) algorithm. We illustrate this approach through two different experiments, one based on solving 8-puzzle configurations, and the other on path planning. In both experiments, subjects were able to speed up the computation of a solution through a reduction of search space in WA*. Our results establish the ability of subjects to intervene in heuristic search progression, with effects which are commensurate to their control of PFC asymmetry: this opens the way to new mechanisms for the implementation of hybrid cognitive systems.

## Introduction

Brain-Computer Interfaces (BCI) have been a major component of human augmentation research (Schmorrow, 2005), in which intelligent processing was harnessed to extend human information processing abilities. However, it has recently been suggested that BCI-based human augmentation could be used to address the problem of human control over autonomous Artificial Intelligence (AI) systems (Kennedy, 2014), which several authors have identified as one of the major societal challenges for the future (Kurzweil, 2005; Bostrom, 2014).

The research presented in this article explores new techniques through which the mental attitudes of users could be used to influence, in a principled way, the computational behavior of an AI system using a BCI. Such a research program should aim at endowing humans with high-level control abilities sufficient to steer the flow of AI computations, irrespective of their low-level details, while preserving an understanding of the AI computation’s goal.

One mechanism that emerged as a good candidate to bridge the gap between cognitive mechanisms and AI systems is heuristic functions, which have a long history in cognitive science and AI. Recently, a new vision of heuristics has emerged within AI, one which emphasizes pruning as the most important function of heuristics, rather than providing information guiding towards an optimal solution (Sturtevant et al., 2012). This suggests that users’ cognitive attitudes could have an input at the level of heuristic functions calculation, hence influencing the amount of pruning associated with a heuristic. In turn, such pruning during search might speed up reaching a solution, at the risk of selecting a suboptimal one. We can thus hypothesize that cognitive attitudes related to motivation, reward anticipation, or risk acceptance, are candidates to control heuristic search.

There is a rich body of literature which suggests that these cognitive states are correlated to Prefrontal Cortex (PFC) asymmetry (Davidson et al., 1990; Sutton and Davidson, 1997; Santesso et al., 2008; Gorka et al., 2015), which can be quantified using EEG or fNIRS and used as a neural marker of the above states. In addition, frontal asymmetry has been shown to be controllable through Neurofeedback (NF) in clinical applications (Rosenfeld et al., 1995; Baehr et al., 2001; Zotev et al., 2014), a property we have used for the development of affective BCI (Gilroy et al., 2013). There has also been significant previous research in the use of fNIRS for BCI (Solovey et al., 2009), including the measurement of task difficulty in conjunction with computer gameplay (Girouard et al., 2009), or as an additional input channel to interactive systems (Solovey et al., 2012). This research has, however, primarily investigated fNIRS for passive BCI. Finally, Doi et al. (2013) have shown fNIRS to be well-suited to the study of emotional responses in the PFC, which is our target area, while other work (Tuscan et al., 2013; Naseer and Hong, 2015) has confirmed the accessibility of specific prefrontal areas to fNIRS.

In this article, we introduce human control of heuristic search behavior based on a BCI, and present results from two fully-implemented early experiments on traditional search problems. The underlying idea is to use PFC asymmetry, captured through fNIRS to control the progression of heuristic search in a principled fashion, using mathematical properties of heuristic search. User active control of their mental disposition is implemented through an fNIRS NF paradigm, in which users receive as feedback visual cues on the heuristic search strategy.

Some of the most significant research in BCI interfacing to AI systems has taken place in the field of BCI-enhanced Information Retrieval. Gerson et al. (2006) have demonstrated increased human performance in satellite image analysis when a BCI system was used to detect regions of interest despite very fast, almost subliminal, visual scanning by users. Kapoor et al. (2008) have used EEG-based BCI in combination with computer vision to improve image categorization. Eugster et al. (2014) have described how BCI could assist to automatically detect term relevance during Information Retrieval tasks. The novelty of our work thus consists in investigating BCI control of basic AI algorithms themselves.

After introducing the various elements of our system, we report experiments carried out on two traditional search problems, 8-puzzle and path planning, for which the automatic search for a solution was influenced through BCI input. Finally, to assess the usability of the BCI, we analyze subjects’ performance on the NF task and its impact on heuristic search behavior for each application.

## Materials and Methods

### A Framework for BCI Control of Heuristic Search

In order to implement BCI interfacing to AI computations, we need to identify a principled mechanism through which user cognitive strategies can influence the performance of AI algorithms. Since heuristic search remains at the heart of many AI computations, we have designed a specific mechanism to influence heuristic search parameters.

Heuristic search algorithms, such as A*, provide optimal solution to search problems through the use of a heuristic function (Pearl, 1984). However, it is possible to tune the behavior of heuristic search towards more efficient computations using the precision-complexity exchange property (Pohl, 2010), which allows to produce solutions faster when relaxing strict solution optimality requirements. One such method, introduced by Pohl (1970), consists in redefining A*’s standard heuristic function as a weighted formula of cost *g(n)* and heuristic estimate *h(n)*:

(1) f(n) = (1−w)×g(n)+w×h(n)

where the weighting coefficient *w* is dynamically altered during the search process itself (Pearl, 1984; Hansen and Zhou, 2007). This approach can also be shown to be ∈-admissible, meaning that any deviation from the optimal solution introduced by dynamic weighting is bound by an ∈ factor (precision). The weighting coefficient *w* will thus serve as the main implementation mechanism to influence AI computations and will be the target of the BCI signal. Thereafter, we will refer to this approach as Weighted A* (WA*) with dynamic weighting (Ebendt and Drechsler, 2009).

Our WA* implementation is based on the comprehensive A* description given in Pearl (1984), which we have modified to support various options for dynamic weighting; one of these supports changes in dynamic weighting at various stages of search progression, defined in terms of percentage of maximum node expansion.

We will be using two traditional heuristic search problems to support our experiments: the 8-puzzle, and grid-based path planning. The entire solution set of the 8-puzzle has been studied by Reinefeld (1993), who has generated all 9!/2 solvable tile configurations, computed optimal solutions for all problem instances, and identified specific configurations according to their number of solutions. We have selected as runtime examples for our experiments the two 8-puzzle configurations with the greatest number of solutions (64). The rationale for also experimenting with path planning is that, unlike the 8-puzzle whose search space is abstract, it supports direct visualization of search space progression, which is obviously of relevance when investigating a NF paradigm for BCI.

The main mechanism through which WA* affects search performance is a reduction in the number of nodes expanded during search (Hansen and Zhou, 2007), which is a convenient way to present the impact of dynamic weighting. In their study of various benchmarks, Wilt and Ruml (2012) plotted the number of expanded nodes as a function of the weighting coefficient under a histogram format, which we will adopt to present experimental results (see “Results and Discussion” Section).

**Figure 1:**
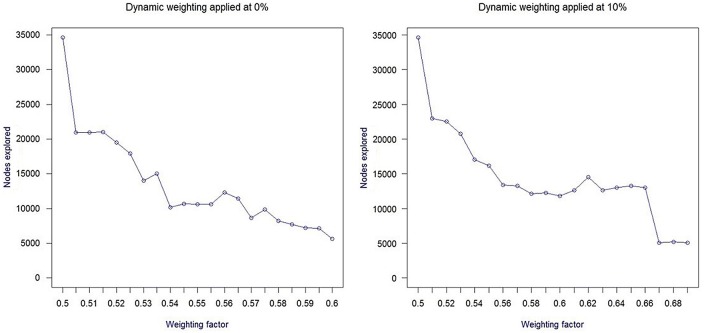
**Impact of dynamic weighting on the size of the 8-puzzle search space**.

In order to determine the optimal variation range for dynamic weighting, we have explored the impact of various values of the weighting coefficient on the number of nodes explored (see Pearl and Kim, 1982). Our results confirmed that for the 8-puzzle problem set (using Manhattan distance as the baseline heuristic function), the most significant performance improvement takes place for dynamic weighting in the *w* = [0.5; 0.7] range. Figure 1 depicts the variation of search space as a function of dynamic weighting, also considering the timing of dynamic weighting modification.

In a similar fashion, offline simulations of our WA* implementation for path planning have shown that the maximum variation in path planning performance is observed for the weighting interval [0.5; 0.6]^1^, using straight line distance as the baseline heuristic function.

From a user’s cognitive perspective, seeing a solution appearing faster can be associated to motivational aspects, and there is indeed abundant research that associates PFC asymmetry to motivational aspects and reward anticipation (Gorka et al., 2015) under the high-level dimension of approach (Sutton and Davidson, 1997). The latter point is further supported by the identified role of the DLPFC in reward encoding modulation, considering the correlation between DLPFC asymmetry and approach. Amodio et al. (2004) have analyzed the correlates of PFC asymmetry from a regulatory perspective. More specifically, they found the approach regulation to be most relevant to “pre-goal states”, during which efforts are mobilized towards the goal. As a result, they associate frontal activity with goal dispositions, and the behavioral aspects of goal pursuit. This should be relevant to any search-based problem solving application with the solution as a goal.

Hence, the overall system can be described as follows: (i) the level of PFC asymmetry will serve as the BCI signal acquired through fNIRS; (ii) the user, as a cognitive strategy, will express their anticipation of search progression, which in turn will affect PFC asymmetry in a NF setting; and (iii) the real-time variation of PFC asymmetry will be mapped onto the weighting factor of WA*, thereby determining the precision-complexity tradeoff and speeding up the reach for a solution.

The above BCI approach is based on a mapping function that determines the optimal variation of dynamic weighting as a function of variations of PFC asymmetry. Considering results from offline simulations (Figure 1), the variation in performance can be roughly approximated by a linear function over most of the range, which supports the definition of a linear mapping function from the BCI asymmetry score to the weighting coefficient.

The second aspect of mapping is to decide at which stage of search progression dynamic weighting should be triggered. For our 8-puzzle application, we have decided to modify *w* only once, at an early stage of search progression, the exact timing depending on the temporal variation pattern of PFC asymmetry. During preliminary tests, we found the impact of dynamic weighting to be most significant when applied between 0% (onset) and 25% of the search progression (defined as the number of expanded nodes) as illustrated in Figure 1 for a dynamic weighting interval of [0.5; 0.7].

The requirements on the timing of dynamic weighting are less stringent for path planning. This is why we have allowed modifications to w at regular interval throughout the search process. The weighting coefficient is updated at regular intervals (2 Hz) during the search process based on the sampling of the fNIRS PFC asymmetry value. However, to avoid erratic behavior that would result from varying *w* in opposite directions during a NF epoch, mapping operates by only increasing *w*, so that the current value corresponds to the maximum value over time.

Finally, we ensured that the timescale of WA*-based solution computation should be compatible with the duration of NF epochs in order to enable appropriate user experiments. This is mostly relevant to path planning, because of the repeated updating of the weighting coefficient. The computation of a solution path on our 40 × 60 grid takes on average 45 s on our hardware configuration, the search process being slowed down by the I/O exchanges required to display the nodes expanded during search as well as additional timing loops calibrated to the target total duration.

In “Experiment I: BCI Control of 8-Puzzle Solving” Section, we describe the design of our fNIRS-based BCI system, as well as the NF protocol that has been used in two experiments, each one using a different search problem (8-puzzle and path planning).

### Apparatus

We used a Biopac Systems fNIR400 optical brain imaging device with a 16-channel sensor (fixed 2.5 cm source detector separation) to operationalize real-time BCI input and record brain-activity data for *post hoc* analysis (see Ruocco et al., 2014 for channel locations). Raw data and oxygenated-hemoglobin (HbO) values^2^ were collected with 2 Hz sampling rate (using COBI Studio) and was sent to bespoke client experimental software over TCP/IP (using FnirSoft v3.5 DAQ Tools). We followed the recommendations of Solovey et al. (2012) for using fNIRS in Human-Computer Interaction settings to inform the experimental set-up. Subjects were seated approximately 47″ (1.2 m) away from a 24″ flat monitor, in a comfortable chair to help minimize movements. The room was quiet (but not soundproof) and dimly lit, and the sensor band positioned on the subjects’ forehead was also covered with non-transparent fabric to block out ambient light. Subjects were instructed to refrain from moving (particularly their limbs and head), talking and frowning while carrying out experimental tasks.

### Experimental Paradigm

For both experiments, we applied the same generic set-up, with differences in the design of the experimental tasks and methods. Details of these will be presented in the subsequent sections. Subjects were right-handed, reported no treatment history for psychiatric conditions and provided written consent prior to participation. The experiment was approved by a research ethics committee at the authors’ institution. Subjects were compensated with an online retailer voucher equivalent to $30.

HbO for each sensor channel was calculated with respect to a baseline measured at the beginning of each block (Ayaz et al., 2010). We derived a real-time metric of left-asymmetry by averaging HbO values over the four leftmost and four rightmost channels (located over the left and right dorsolateral PFC, respectively), and subtracting Right from Left (Cavazza et al., 2015; also see Doi et al., 2013). The resulting asymmetry metric reflects inter-hemispheric difference in HbO change, in micromolar units (μM/L; see Aranyi et al., 2015 for a detailed description of brain-signal selection and integration for the current experimental tasks). In both experiments, data was collected in a set of identical blocks consisting of a set of epochs (short time periods with a specific task).

## Experiment I: BCI Control of 8-Puzzle Solving

### Neurofeedback Protocol

**Figure 2:**
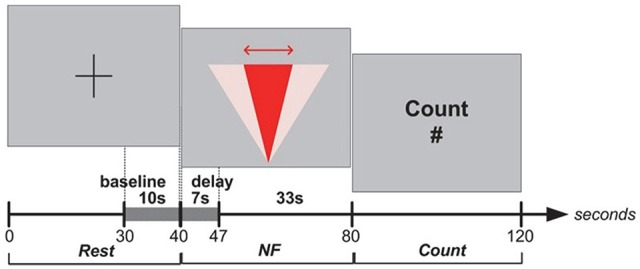
**Experiment I: Rest, Neurofeedback (NF) and Count epochs**.

**Figure 3:**
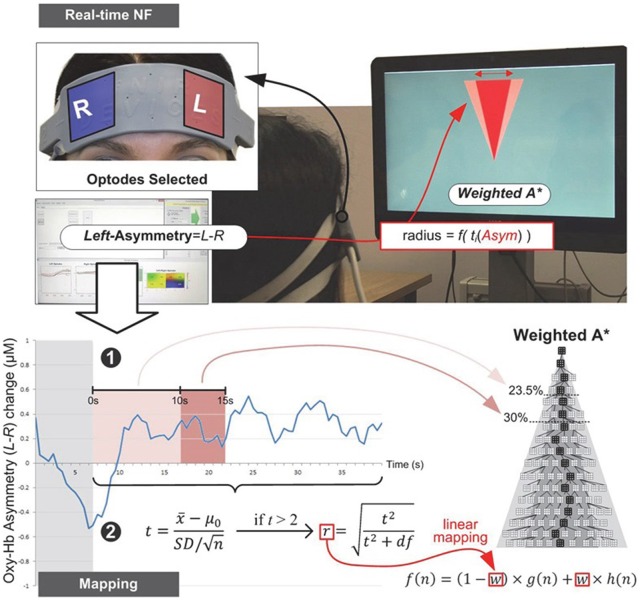
**Experiment I—Subjects equipped with an fNIRS sensor engage in a NF task whose visual display is a metaphor for the search space they are trying to reduce.** Mapping between fNIRS signal and the WA* weighting coefficient is implemented via real-time statistical testing of prefrontal asymmetry. The weighting coefficient is only modified once, with several attempts in the early stages of the search process. Note that since each epoch (without the first 7 s, shown in gray) contained at least 66 observations (33 s with 2 Hz sampling frequency), we applied as threshold criterion the *t* critical value for *p* = 0.05 (two-tailed) with 65° of freedom, *t*_crit (65)_ = 2.00.

In Experiment I, blocks were structured as the following sequence of epochs: Rest, NF and Count (see Figure 2). Each epoch lasted 40 s. The last 10 s of each Rest epoch was used to calculate the baseline for the block. Rest epochs contained no specified cognitive tasks and no feedback on prefrontal asymmetry, and they were used to calculate the baseline for successive blocks; therefore, Rest epochs were not analyzed. During the NF epoch, subjects were instructed to use their thoughts to narrow a red cone presented on the screen (Figure 3), which corresponds metaphorically to the focus of the (automatic) search process. They were not instructed to think about a solution or to try to solve the puzzle themselves (most of them were actually not familiar with the 8-puzzle problem). They discussed their strategies with the experimenter after each practice block (three in total), and reported their cognitive strategy after each experimental block (six in total). Because the hemodynamic response measured by fNIRS occurs in approximately 7 s (see Bunce et al., 2006), the first 7 s of data in each NF and Count epoch were not analyzed (see Figure 6A). Success in each NF epoch was determined by performing a one-sample *t*-test on the asymmetry scores collected during the epoch against the test value of 0, upon the completion of the epoch. This tested whether there was a statistically significant increase in asymmetry against asymmetry recorded during the baseline (measured during Rest), since the mean of prefrontal asymmetry during baseline was defined as the zero point (regardless of the actual level of asymmetry during baseline measurement). If this increase was statistically significant, the effect-size measure^3^ *r* was calculated to characterize the magnitude of difference from the baseline. We included a Count epoch lasting for 40 s following NF, during which subjects were instructed to mentally count backwards from 100 by subtracting a given integer (3, 4, 6 or 7). This mental counting task is theoretically unrelated to left-asymmetry and it is one of the most commonly used prefrontal activities in fNIRS based BCI research (Naseer and Hong, 2015). The Count epoch was included to distract subjects’ attention from the cognitive strategies they used during NF (see Zotev et al., 2011), and to promote prefrontal activation converging to baseline after NF.

## Experiment II: BCI Control of Grid-Based Path Planning

Although grid-based path planning is another traditional testbed for heuristic search algorithms (Pearl, 1984), it differs from the 8-puzzle in that it allows a direct visualization of search space and search progression, because of its inherent spatial nature. We have thus designed a second experiment to explore how visualizing the early progression of the search process would affect NF.

We have opted for a relatively simple and symmetrical obstacle configuration made of tiled obstacles perpendicular to the main axis from the start cell to the goal cell. In this configuration, WA* dynamic weighting will tend to focus the search space, thereby speeding up the computation of a solution path.

For consistency, we have preserved the dynamic cone used in the 8-puzzle version as a visual feedback channel: although in this case the cone is more directly related to search space progression, it provides a geometrically simpler feedback in case the actual search space displayed grows into irregular patterns; the cone is simply overlaid on the actual grid, aligned on the progression axis, with the large section of the cone towards the goal cell.

The mapping function is designed from the offline calibration of path planning using WA* and the observed PFC asymmetry scores. Preliminary offline simulations with WA* alone have shown that the maximum variation in path planning performance is observed for the weighting interval [0.5; 0.6]^4^, while the asymmetry score (after baseline) varies in the [0; 1.1] interval. We have opted for a linear mapping approach, which gives the linear mapping formula:

(2) $$\text{w}\;=\;\frac{1}{11}\times \text{Asym}+0.5$$

### Neurofeedback Protocol

Because we used our results from Experiment I to design Experiment II, block design, data pre-processing and analysis differ in a number of respects between the two experiments. These differences are described below, and summarized in Table 1.

**Table 1:** **Summary of differences in block design between the experiments**.

	Experiment I	Experiment II
Practice	3 blocks	1 block
NF blocks	6	8
Baseline task	Rest	Count
Success test	Parametric	Bootstrapping
Test timing	Real-time	*Post hoc*
Filtering	None	SMAR, FIR

**Figure 4:**
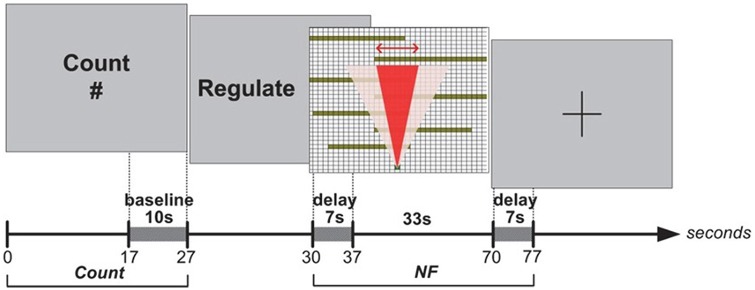
**Experiment II: Count and NF epochs.** Left-asymmetric increase in DL-Prefrontal Cortex (PFC) activity during NF was mapped to the width of the red cone, which was used as a visual metaphor (i.e., narrowing the beam of a searchlight) for supporting the search process.

**Figure 5:**
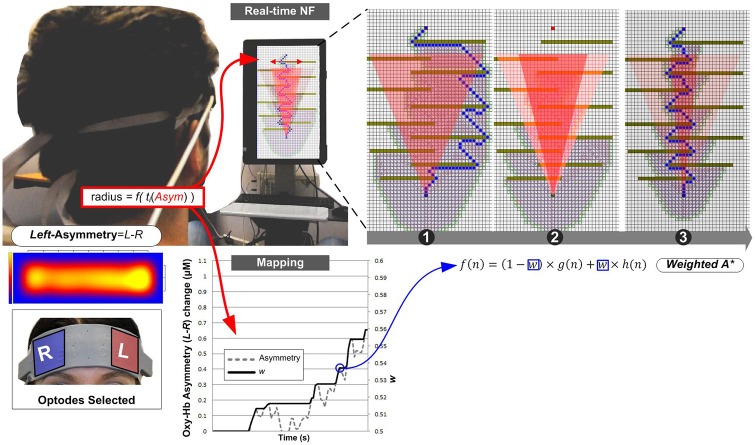
**Experiment II—Subjects equipped with an fNIRS sensor engage in a NF task whose visual display is this time the progression of the search process in path planning on a grid.** The real-time mapping shown on the graph illustrates how increasing *w* values depends on positive up-regulation of asymmetry score. Unlike Experiment I, the weighting coefficient can be subject to successive dynamic increases during search. Examples: (1) shows a standard un-influenced solution path; (2) shows details of the cone visual feedback to the user’s positive input, matching to the acceleration of the search process, thus to a reduction of the search space (nodes unexplored on the right-hand side); and (3) shows the alternative solution produced by WA* under the subject’s influence.

In Experiment II, each block started with an epoch lasting for 27 s, during which subjects were instructed to carry out a simple mental counting task (counting backwards from 500 by a given integer) while looking at the computer screen. Baseline for the block was measured during the final 10 s of this epoch (see Figure 4). This counting task was used to control for unwanted mental processes (see Sarkheil et al., 2015) while measuring baseline, because it is unrelated to asymmetry, and Experiment I indicated that it is comparable in subjective difficulty to the NF task. Following baseline, subjects received a visual prompt (3 s) to abandon the counting task and regulate their brain activity to influence the path-planning algorithm, during which they would receive real-time NF of their brain activity. This NF epoch lasted for 47 s. During the NF epoch, subjects were instructed to mentally express their eagerness to reach a solution, and they were instructed that their brain activity was controlling the width of the cone, which corresponds to the focus of the automatic search process (Figure 5). Once again they were not instructed to think about a solution themselves. Because these instructions were more suggestive of potentially successful thinking strategies (i.e., expressing eagerness), and subjects also received feedback on search progression and the target state, this protocol only included a single practice block (after which they discussed their thinking strategy with the experimenter), and 8 experimental blocks (after each block they reported their thinking strategies). To compensate for the approximate 7 s delay in hemodynamic response, the algorithm was left on stand-by for the first 7 s of the NF epoch and the feedback channel remained unchanged while subjects were already applying their cognitive strategies. These 7 s were not considered in *post hoc* analyses. We characterized NF success as a statistically significant increase in left-asymmetry compared to baseline (defined as the zero point) during the NF epoch within a block. We determined block success *post hoc*, using one-sample *t*-tests with bootstrapping (1000 samples, 95% confidence intervals) on asymmetry values collected during the NF epoch against the test value 0. Furthermore, we calculated the effect-size measure *r* to characterize the magnitude of increase in asymmetry in successful blocks (see Cavazza et al., 2015). Since we calculated block success *post hoc* in Experiment II, we could include a filtering process which allowed for identifying channels as problematic using sliding-window motion artifact rejection (Ayaz et al., 2010). To attenuate noise, data was also low-pass filtered using a finite impulse response (FIR) filter with order 20 and 0.1 Hz cut-off frequency (Ayaz et al., 2010).

## Results and Discussion

### Experiment I

Eleven adult subjects participated in Experiment I (Age: *M* = 37.18 years, SD = 11.21, range = 20–52; 3 female). Out of all 66 blocks completed by the 11 subjects, 38 (58%) contained an NF epoch with statistically significant left-side asymmetry; these blocks were considered successful^5^. Each subject had at least one successful block, and eight subjects (73%) had at least three successful blocks (i.e., half of blocks successful). No subject achieved NF success on all six blocks.

**Figure 6:**
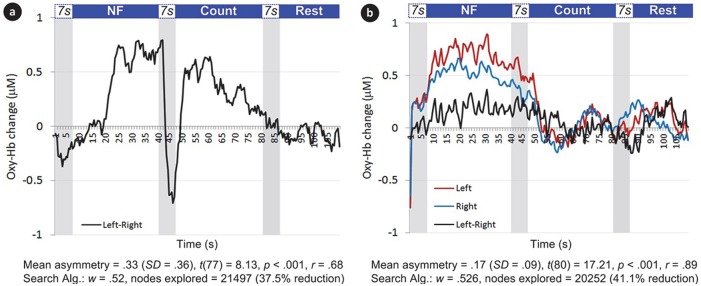
**Examples of average left and right oxygenated-hemoglobin (HbO) changes over time, as well as asymmetry, during two successful blocks (a, b) in Experiment I.** Areas in gray represent the first 7 s of each epoch (i.e., the approximate delay of the hemodynamic response). Note that during the NF epoch, HbO increases bilaterally, with asymmetry to the left; during the Count epoch following NF, HbO decreases on both sides; during Rest, HbO further decreases towards baseline.

Since fNIRS signals are relative values, it can be difficult to compare them across subjects (Sakatani et al., 2013); moreover, the magnitude of oxygenation changes can also differ substantially across blocks within the same subject. Our mapping strategy was designed to mitigate the issue of comparability. We demonstrate this through two examples of a successful block from two different subjects (see Figure 6).

Figure 6A shows a larger mean asymmetry during the NF epoch than Figure 6B (ΔOxy-Hb = 0.33 and 0.17, respectively); however, the dispersion of asymmetry scores during the epoch was also larger compared to the mean (SD = 0.36 and 0.09, respectively). In other words, left-side oxygenation was more consistently above right-side throughout the NF epoch in Figure 6B; consequently, the *t* value was larger (*t* is calculated using the mean, standard deviation and number of data points), leading to a larger *r* value (*r* is based on *t* and the degrees of freedom), mapped to a larger *w* value, leading to greater reduction in search space (see Equation (2) for formulae).

We only calculated *r* values for epochs where left-side asymmetry was statistically significantly above 0 (i.e., the NF signal was used to influence the algorithm only when there was statistical evidence for left asymmetry). The lowest *r* value (0.28) shows that we could reliably detect medium (and large) effect sizes (*r* ≥ 0.30) during 40 s-long NF epochs with 2 Hz sampling frequency. Although several NF epochs approached the maximum *r* value of 1, which determined the maximum of the dynamic weighting of the search algorithm, the distribution of scores demonstrates that differential weighting was successfully applied based on the *r* effect-size measure. Successful NF epochs result in a commensurate modification of the weighting coefficient *w* via the mapping process, which in turn accelerates search by reducing the search space explored. In this experiment, the weighting coefficient was modified as soon as an *r* value was obtained. However, testing was performed at several early stages of search, meaning that not just *r* magnitude but also the speed at which asymmetry was reached, had an influence on the search process.

**Figure 7:**
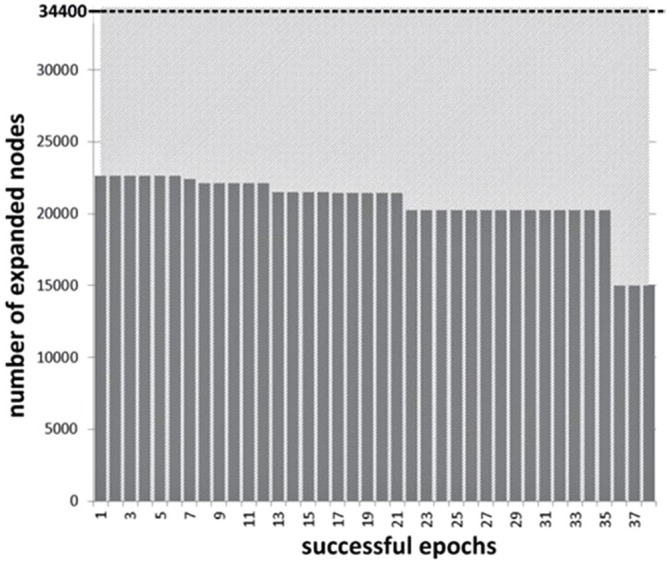
**Experiment I: decrease of the number of the nodes expanded during the state-space search across successful NF epochs (total number of nodes explored without intervention is 34,400 for the 8-puzzle configuration used in the experiments)**.

The reduction in search space for successful NF blocks is presented in Figure 7. Average reduction in nodes visited across the 38 successful NF epochs was 39.5% (SD = 5.65) from the baseline, which has a significant effect for an algorithm of exponential complexity such as WA*. Overall, these findings support the validity of our approach to defining NF success and mapping it to the behavior of the search algorithm. In addition, variations of the BCI signal, within and across subjects, result in different levels of performance improvement for heuristic search. While this is an essential property to envision actual control of heuristic search from users’ cognitive attitude, variation across successful epochs is still moderate in this first experiment.

### Experiment II

Sixteen subjects participated in Experiment II (5 female; Mean age = 34.31 years, SD = 11.31, Range = [21; 62]). They signed the same consent form and received the same compensation as subjects for the first experiment. Data from two subjects had to be discarded (due to technical problems or deviating from instructions), leaving data from 14 subjects to be interpreted, corresponding to 112 blocks. Of these, 53 blocks (47%) were successful (had average asymmetry significantly larger than 0 during NF), using similar criteria as those defined for the first experiment, which are valid for both applications as they measure NF success^6^. At subject level, using the same success criterion as above (50% successful blocks), 8 subjects out of 14 were successful (57%).

**Figure 8:**
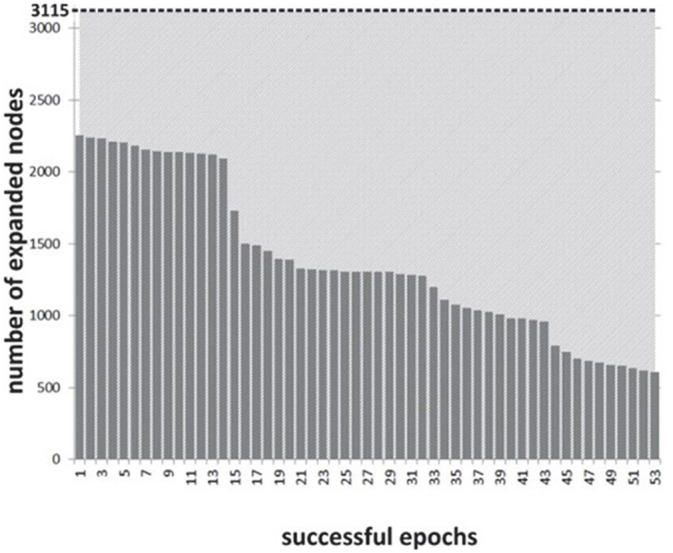
**Experiment II: number of nodes expanded during state-space search across successful NF epochs**.

The distribution of search time reduction across blocks shows a range of effects (see Figure 8), commensurate to the distribution of asymmetry values across blocks and subjects. The overall time reduction is less marked than with the 8-puzzle application: this is due to lower NF scores, but only in part, as the scope for search space reduction was more significant in the 8-puzzle example configuration than in the specific path planning one.

**Figure 9:**
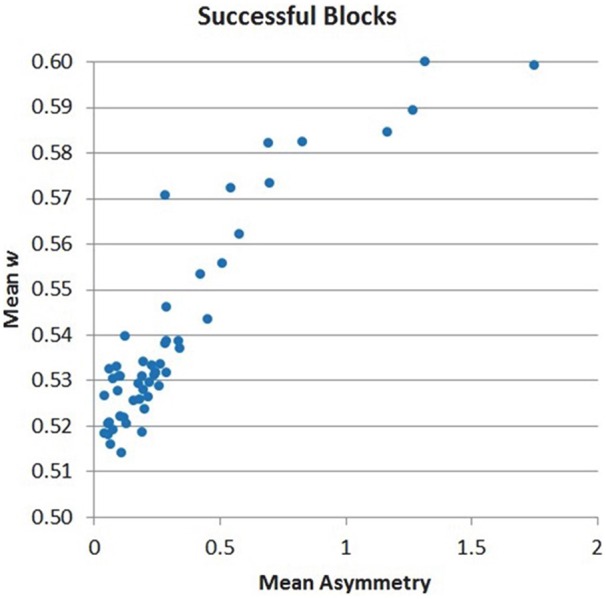
**Distribution of *w* values across successful NF blocks (*r* = 0.895, *p* < 0.001)**.

The distribution of *w* across samples is suggesting that the chosen mapping allows a progressive effect in speeding up search based on the level of PFC asymmetry, rather than a binary effect (Figure 9). This finding, already observed for the 8-puzzle application, is again an important validation for BCI design, as it supports the use of the BCI signal to progressively control AI search behavior (within and across subjects) rather than behaving as an all-or-nothing switch.

Search space reduction is particularly marked for values of *w* > 0.55 in the specific layout tested. We have isolated the most successful blocks and studied them specifically. Overall, a typical successful block will see a rapid onset of PFC asymmetry and will result in a runtime reduction of 50% or more, through a reduction of the search space (Figure 8), runtime reduction being the most visible effect for the subject across trials. In this second experiment, the spread of search space reduction (number of nodes) is much greater across successful blocks and would make possible a finer mapping between asymmetry values and speeding up of the search process.

We collected post-experiment user feedback on the actual cognitive strategies they used for NF: it highlighted a mix of through contents related to both approach and positive valence, apparently influenced by the visual nature of the application. Users reported various sorts of mental “cheering” as if encouraging runners during a race, or imagining taking part in the race themselves, as well as the use of more abstract thinking strategies to generate a feeling of eagerness, such as reminiscence of appetitive stimuli or pleasant memories.

**Figure 10:**
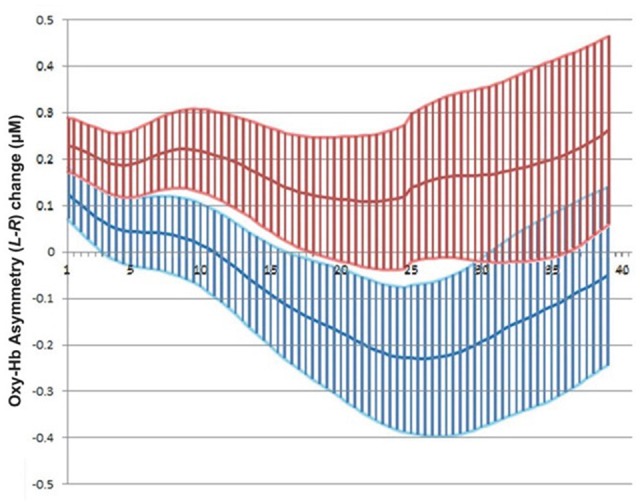
**Mean and standard error of fNIRS signal across all successful blocks in Experiment II for left (Red) and right (Blue) sides separately (left rises above right during NF epoch)**.

The overall NF success scores for both experiments differed slightly (58%, in Experiment I, vs. 47%, in Experiment II, on a block basis), suggesting that the visibility of actual search data in path planning did not improve subjects’ performance over the use of an abstract representation as a visual feedback channel (Figure 10). Potential explanations include the fact that such real-time data can actually appear distracting because of their perceived spatial complexity or, in the specific case of path planning the real-time visualization of back-tracking steps, which would appear uncanny as visual feedback, distracting the users from the main visual feedback target which is the overlaid cone. It is important to consider overall performance strictly in the context of previous NF work, in particular taking into account the absence of any significant training in our experiments. Previous NF experiments reported in clinical research have included extensive training spread over multiple sessions: for instance, Rosenfeld et al. (1995) reported training subjects over 3 days prior to NF experiments, and Kotchoubey et al. (2002) up to eight weeks. We have conspicuously refrained from training subjects over repeated sessions, due to the possible mood alterations induced by prefrontal asymmetry NF: the above studies (Rosenfeld et al., 1995; Kotchoubey et al., 2002) took place in a clinical setting while for our experiments, ethical approval did not cover potential long-term effects, leading us to exert caution (even if mood alteration following left-asymmetry training was assumed to be positive, according to the established literature).

In terms of acceleration of the search process, the average reduction in computation time was actually larger with the 8-puzzle experiment, but this can be considered a property of the search space itself, and the fact that weighting coefficient modification tends to have maximum impact if performed early in the search process. The 8-puzzle search process was in that respect easier to control while path planning is highly dependent on obstacle density and layout.

## Conclusion

We have presented a novel use of BCI, aiming at interfacing directly at the algorithmic level of AI computations, supported by a proof of concept experiment on two traditional heuristic search benchmarks. To the best of our knowledge, this work still constitutes the first fully implemented “BCI to autonomous AI” interface of the type advocated by Kennedy (2014).

These early results establish the possibility for high-level cognitive disposition to influence, in a principled fashion, the behavior of basic AI algorithms, which despite their elementary nature stand at the heart of many complex AI systems. For instance, the same basic heuristic search mechanisms that we have studied here are central to heuristic search planning (Bonet and Geffner, 2001) which can be applied to the resolution of complex, real-world problems. This suggests that the fundamental mechanisms we have introduced here could be transposed to more complex AI systems more representative of those that would require human supervision in the long term.

However, our results have also revealed several limitations in the design of our experimental protocol. More attention should be dedicated to facilitating users’ cognitive strategies, especially since most subjects were unfamiliar with the type of problem solving they were meant to control. In particular, one difficulty faced by subjects was to control approach in a rather emotionally neutral context, which contained no appetitive stimuli traditionally associated with the approach, possibly due to the rather abstract nature of the task and the absence of real-world reward. This may contribute to lower success scores than in our previous experiments using fNIRS NF, where subjects expressed affective disposition towards virtual characters (Aranyi et al., 2016). In terms of experiments interpretation, a more specific design should include additional validation of cognitive dispositions, e.g., through selective questionnaires: this will help ensuring the specific nature of DLPFC activation.

Finally, although the observed NF success scores are compatible with the state-of-the-art in the absence of extensive subject training, further validation should instead aim at maximizing success scores in the presence of NF training, also addressing the issue of cognitive strategies.

## Ethics Statement

The study was approved by Teesside University’s Research Ethics Committee. In the consent form, the purpose of the research and the procedure of the experiment were fully outlined to the participants, as well as the risks and benefits, and confidentiality procedures.

## Author Contributions

MC conceived and designed the study, and developed part of the planning software. GA conducted the experiments and collected the data, as well as produced the data analysis. FC conducted the experiments and developed the path-planning and other supporting software. MC, GA and FC wrote the article.

## Conflict of Interest Statement

The authors declare that the research was conducted in the absence of any commercial or financial relationships that could be construed as a potential conflict of interest.

## Footnotes

^1^Note that this differs from the optimal variation range for the weighting coefficient in our 8-puzzle experiment.

^2^Although we used changes in oxygenated hemoglobin (HbO) values for BCI input as it is commonly more associated with neural activity (Ruocco et al., 2014), deoxygenated (HbR) and total (HbT) hemoglobin measurements were also collected.

^3^The effect-size *r* is constrained between 0 and 1 (see Figure 3 for calculation). According to Cohen (1988) conventions, *r* = 0.10 corresponds to small, 0.30 to medium, and 0.50 to large effect-size.

^4^Note that this differs from the optimal variation range for the weighting coefficient in our 8-puzzle experiment.

^5^Note that in Experiment I, NF success was determined real-time by the experimental software by running *t*-tests on asymmetry scores after the completion of each epoch. The assumption of normality was not tested by the experimental software; however, *post hoc* analyses using bootstrapping resampling method on a subset of epochs resulted in accepting the same epochs as successful. Additionally, *post hoc* correction for family-wise error yielded the same result, with the exception of one single epoch.

^6^Block success in Experiment II was determined *post hoc* using one-sample *t*-tests on asymmetry values collected during the NF epoch (40 s sampled at 2 Hz frequency) against the test value 0, with bootstrapping resampling method (1000 samples, 95% confidence intervals).
